# Supplementary material for: Comparative Research on Metabolites of Different Species of Epichloë Endophytes and Their Host Achnatherum sibiricum
Source: J Fungi (Basel). 2022 Jun 10;8(6):619. doi: 10.3390/jof8060619 (PMC9225340; doi:10.3390/jof8060619)
Supplement: Supplementary file 1 [file jof-08-00619-s001.zip › jof-1702914-supplementary.pdf]

Table S1. Metabolites identified in mycelium of *E. gansuensis* (Eg) and *E. sibirica* (Es) from GC–MS analysis. The quantities are showed with "relative content ± standard deviation".

Class	Compound	Relative content	
		Eg	Es
Amino acid	Ser	0.0100 ± 0.0017	0.0105 ± 0.0026
Amino acid	Thr	0.0142 ± 0.0015	0.0143 ± 0.0018
Amino acid	Gly	0.0058 ± 0.0012	0.0054 ± 0.0010
Amino acid	Hse	0.0043 ± 0.0007	0.0047 ± 0.0012
Amino acid	Pro	0.0913 ± 0.0081	0.0540 ± 0.0085
Amino acid	Asp	0.0314 ± 0.0047	0.0328 ± 0.0094
Amino acid	Glu	0.0152 ± 0.0013	0.0127 ± 0.0006
Amino acid	Asn	0.0074 ± 0.0008	0.0075 ± 0.0015
Amino acid	Lys	0.0136 ± 0.0011	0.0137 ± 0.0013
Amino acid	Tyr	0.0050 ± 0.0010	0.0101 ± 0.0016
Amino acid	Phe	0.0357 ± 0.0033	0.0388 ± 0.0048
Sugar	Fructose	0.0022 ± 0.0004	0.0024 ± 0.0005
Sugar	Galactose	0.0098 ± 0.0016	0.0104 ± 0.0022
Sugar	Talose	0.0056 ± 0.0006	0.0055 ± 0.0008
Sugar	Trehalose	1.0774 ± 0.0707	1.4133 ± 0.0531
Sugar	Mannobiose	0.0034 ± 0.0005	0.0034 ± 0.0005
Sugar alcohol	Glycerol	0.1139 ± 0.0138	0.1135 ± 0.0155
Sugar alcohol	Threitol	0.0066 ± 0.0009	0.0069 ± 0.0010
Sugar alcohol	Erythritol	0.0062 ± 0.0008	0.0065 ± 0.0009
Sugar alcohol	Arabitol	0.0277 ± 0.0024	0.0283 ± 0.0027
Sugar alcohol	Mannitol	1.5818 ± 0.0990	1.6271 ± 0.0920
Sugar alcohol	Inositol	0.0274 ± 0.0031	0.0292 ± 0.0017
Sugar alcohol	Sorbitol	0.0700 ± 0.0040	0.0707 ± 0.0070
Organic acid	Butanedioic acid	0.0373 ± 0.0028	0.0379 ± 0.0028
Organic acid	Erythronic acid	0.0045 ± 0.0005	0.0053 ± 0.0009
Organic acid	Threonic acid	0.0064 ± 0.0005	0.0057 ± 0.0010
Organic acid	Ribonic acid	0.0043 ± 0.0006	0.0047 ± 0.0007
Fatty acid	Dodecanoic acid	0.0048 ± 0.0008	0.0050 ± 0.0006
Fatty acid	Tetradecanoic acid	0.0120 ± 0.0012	0.0119 ± 0.0020
Fatty acid	Pentadecanoic acid	0.0040 ± 0.0006	0.0043 ± 0.0007
Fatty acid	Hexadecanoic acid	0.2394 ± 0.0110	0.2498 ± 0.0164
Fatty acid	Heptadecanoic acid	0.0048 ± 0.0005	0.0049 ± 0.0007
Fatty acid	trans-9-Octadecenoic acid	0.0253 ± 0.0017	0.0338 ± 0.0033
Fatty acid	α-Linolenic acid	0.0322 ± 0.0020	0.0529 ± 0.0028
Fatty acid	Octadecanoic acid	0.2708 ± 0.0273	0.2731 ± 0.0079

Table S2. Metabolites identified in leaves of *A. sibiricum* infected with *E. gansuensis* (Eg+), *E. sibirica* (Es+) and endophyte-free (E−) plants from GC–MS analysis. The quantities are showed with "relative content ± standard deviation".

Class	Compound	Relative content		
		E−	Eg+	Es+
Amino acid	Thr	0.0214 ± 0.0034	0.0222 ± 0.0033	0.0220 ± 0.0027
Amino acid	Gly	0.0051 ± 0.0005	0.0036 ± 0.0006	0.0041 ± 0.0003
Amino acid	Pro	0.0331 ± 0.0065	0.0320 ± 0.0029	0.0359 ± 0.0023
Amino acid	Asp	0.0398 ± 0.0022	0.0390 ± 0.0017	0.0402 ± 0.0047
Amino acid	Orn	0.0069 ± 0.0004	0.0047 ± 0.0004	0.0048 ± 0.0007
Amino acid	Asn	0.1671 ± 0.0095	0.1786 ± 0.0058	0.1785 ± 0.0117
Amino acid	Tyr	0.0048 ± 0.0004	0.0153 ± 0.0021	0.0221 ± 0.0027
Amino acid	Phe	0.0756 ± 0.0053	0.0950 ± 0.0045	0.0982 ± 0.0151
Amino acid	Val	0.0068 ± 0.0004	0.0055 ± 0.0003	0.0050 ± 0.0005
Amino acid	Glu	0.0043 ± 0.0004	0.0043 ± 0.0003	0.0042 ± 0.0005
Amino acid	Ser	0.0044 ± 0.0004	0.0043 ± 0.0003	0.0043 ± 0.0003
Sugar	Fructose	0.3100 ± 0.0208	0.5333 ± 0.0322	0.5600 ± 0.0317
Sugar	Glucose	0.2459 ± 0.0268	0.4147 ± 0.0147	0.4368 ± 0.0380
Sugar	Allose	0.2652 ± 0.0151	0.4160 ± 0.0096	0.4275 ± 0.0274
Sugar	Sucrose	1.3474 ± 0.0819	2.3194 ± 0.0767	2.2509 ± 0.0887
Sugar	Maltose	0.0378 ± 0.0043	0.0637 ± 0.0038	0.1102 ± 0.0095
Sugar	Galactose	0.0236 ± 0.0016	0.0277 ± 0.0043	0.0235 ± 0.0016
Sugar	Melibiose	0.0034 ± 0.0003	0.0036 ± 0.0003	0.0036 ± 0.0002
Sugar	β-Gentiobiose	0.1181 ± 0.0144	0.1539 ± 0.0216	0.1233 ± 0.0113
Sugar	Lyxose	0.0349 ± 0.0015	0.0346 ± 0.0028	0.0336 ± 0.0024
Sugar alcohol	Glycerol	0.0437 ± 0.0036	0.0445 ± 0.0041	0.0497 ± 0.0039
Sugar alcohol	Inositol	0.0595 ± 0.0061	0.0683 ± 0.0069	0.0514 ± 0.0053
Sugar alcohol	Mannitol	–	0.0330 ± 0.0033	0.0343 ± 0.0040
Organic acid	Hexanoic acid	0.0032 ± 0.0004	0.0034 ± 0.0004	0.0035 ± 0.0004
Organic acid	Butanedioic acid	0.4483 ± 0.0228	0.4508 ± 0.0365	0.4435 ± 0.0400
Organic acid	Malic acid	0.0515 ± 0.0057	0.0478 ± 0.0046	0.0542 ± 0.0064
Organic acid	Propanoic acid	0.0130 ± 0.0013	0.0135 ± 0.0012	0.0139 ± 0.0022
Organic acid	Butanoic acid	0.1159 ± 0.0113	0.1237 ± 0.0219	0.1298 ± 0.0160
Organic acid	Threonic acid	0.0036 ± 0.0003	0.0158 ± 0.0017	0.0141 ± 0.0027
Organic acid	Ribonic acid	0.0038 ± 0.0005	0.0065 ± 0.0004	0.0044 ± 0.0004
Organic acid	Sebacic acid	0.0628 ± 0.0047	0.0629 ± 0.0031	0.0683 ± 0.0098
Fatty acid	Tetradecanoic acid	0.0240 ± 0.0014	0.0249 ± 0.0018	0.0249 ± 0.0019
Fatty acid	Hexadecanoic acid	0.4493 ± 0.0170	0.4440 ± 0.0165	0.4532 ± 0.0191
Fatty acid	9,12-Octadecadienoic acid	0.0443 ± 0.0026	0.0453 ± 0.0027	0.0458 ± 0.0026
Fatty acid	α-Linolenic acid	0.1351 ± 0.0123	0.1721 ± 0.0086	0.2211 ± 0.0160
Fatty acid	Octadecanoic acid	0.4776 ± 0.0228	0.3545 ± 0.0148	0.3477 ± 0.0158
Sterol	Stigmasterol	0.0637 ± 0.0028	0.0645 ± 0.0022	0.0646 ± 0.0020
Sterol	β-Sitosterol	0.1585 ± 0.0051	0.1699 ± 0.0171	0.1618 ± 0.0090
Phenols	α-Tocopherol	0.0249 ± 0.0025	0.0343 ± 0.0024	0.0362 ± 0.0010
